# Impact of the Requirement of Bone Mineral Density Evidence on Utilization of Anti-osteoporosis Medications, Clinical Outcome and Medical Expenditures of Patient With Hip Fracture in Taiwan

**DOI:** 10.34172/ijhpm.2020.169

**Published:** 2020-10-03

**Authors:** Chen-Yu Wang, Shau-Huai Fu, Chih-Chien Hung, Rong-Sen Yang, Jou-Wei Lin, Ho-Min Chen, Fei-Yuan Hsiao, Li-Jiuan Shen

**Affiliations:** ^1^School of Pharmacy, College of Medicine, National Taiwan University, Taipei, Taiwan.; ^2^Graduate Institute of Clinical Pharmacy, College of Medicine, National Taiwan University, Taipei, Taiwan.; ^3^Department of Pharmacy, National Taiwan University Hospital Yun-Lin Branch, Douliu, Taiwan.; ^4^Department of Orthopedics, National Taiwan University Hospital Yun-Lin Branch, Douliu, Taiwan.; ^5^Department of Orthopedics, National Taiwan University Hospital, Taipei, Taiwan.; ^6^Cardiovascular Center, National Taiwan University Hospital Yun-Lin Branch, Douliu, Taiwan.; ^7^Health Data Research Center, National Taiwan University, Taipei, Taiwan.; ^8^Department of Pharmacy, National Taiwan University Hospital, Taipei, Taiwan

**Keywords:** Osteoporosis, Osteoporotic Fracture, National Health Insurance, Anti-osteoporosis Medications

## Abstract

**Background:** Since 2011, Taiwan’s National Health Insurance Administration (NHIA) issued a regulation on the reimbursement to anti-osteoporosis medications (AOMs). This study aimed to evaluate the impact of this regulation in reimbursement on the utilization of AOMs, clinical outcomes and associated medical expenditures of patients with incident hip fractures.

**Methods:** By using the National Health Insurance Research Database (NHIRD), patients with incident hip fracture from 2006 to 2015 were identified as our study cohort. Patients younger than 50 years old or prescribed with AOMs within one year prior to incident fracture were excluded. Outcomes of interest were quarterly estimates of the proportion of patients who received bone mineral density (BMD) examination, who were prescribed AOMs, as well as who encountered subsequent osteoporotic fracture-related visits and associated medical expenditures. Particularly, age- and gender specific estimates were reported. An interrupted time series study design with segmented regression model was used to quantitatively explore the impact of the changes of the reimbursement criteria on the level (immediate) and trend (long-term) changes of these outcomes.

**Results: **Our study enrolled 118 493 patients with incident hip fracture with those patients aged older than 80 years old accounting for the largest proportion. A significantly decreased trend of AOMs prescription rates was observed immediately post regulation except for female aged between 65 and 80, while the long-term pattern showed no significant difference. However, the percentage of patients encountered subsequent osteoporotic fracture-related visit was not statistically different between pre- and post-regulation periods. Noteworthy, the policy regulation was associated with an increasing trend of osteoporotic fracture associated medical expenditures, especially for patients older than 80 years old.

**Conclusion: **The regulation on the reimbursement for AOMs decreased the prescribing rate of AOMs immediately although the effect did not sustain thereafter. However, higher subsequent osteoporotic fracture-related medical expenditures were introduced, especially among those very old population.

## Background

**Key Messages**

Implications for policy makers

- This study found that the regulation in reimbursement substantially influenced patients encountered hip fracture, while the impact varied by different age and gender.
- Post regulation, patients aged 80 years and older spent more money on fracture-related expenditures finally, regardless the decreasing prescription rate of anti-osteoporosis medications (AOMs) in the beginning could save National Health Insurance (NHI) expense.
- This study revealed that timely initiation of AOMs post hip fracture was important for patient and economic for National Health Insurance Administration (NHIA), and bone mineral density (BMD) should not be a decisive criteria to the initiation of AOMs therapy.
- The requirement of BMD evidence decreased the prescribing rate of essential AOMs at begining which may save cost, however, introduced higher overall NHI expense in the future.

Implications for the public

This study provided many visualizable and easy to understand information of the impact of health policy on real-world population. In this study, we found that restriction of anti-osteoporosis medications (AOMs) use in the very beginning of incident hip fracture could introduce higher subsequent osteoporotic fracture related cost. Especially for those very old population, a decrease or delay of a proper medication could introduce substantial unwanted impact. Noteworthy, this study also found that almost 75% of patients were not being arranged to received their osteoporosis treatment post their hip fracture. Therefore, patient education of the importance of the pharmacological treatment for subsequent fracture prevention is also crucial to enhance the initiation of AOMs therapy and alleviate the risk and suffering of subsequent fractures.

Osteoporosis and osteoporotic fractures are associated with increased morbidity and mortality as well as a decreased health-related quality of life.^1-3^ In the United States, the direct economic burden of osteoporotic fractures was approximately $17 billion US dollars in 2005 and is projected to increase by 50% in 2025.^4^ There are approximately 536 000 new cases of fragility fractures in 2011 in the United Kingdom, and the economic burden due to new and prior fractures will increase by 24% to 6723 million euro in 2025.^5^ The estimated disability due to osteoporosis is greater than that caused by cancers (except for lung cancer) and high blood pressure related heart disease.^6^

 Osteoporosis also incur significant clinical and economic burden in Taiwan. The results of Nutrition and Health Survey in Taiwan from 2002 to 2008 showed nearly one in two women and one in four men above 50 years old have a low bone mineral density (BMD) and is defined as osteoporosis.^7^ According to our previous study using the National Health Insurance Research Database (NHIRD), there were nearly 337 000 diagnosed osteoporosis patients in 2013, and the incremental change of direct medical costs attributable to osteoporotic fractures were approximately 4000 US dollars per event.

 Fortunately, well-documented evidences demonstrated that anti-osteoporosis medications (AOMs) can significantly reduce the risk of osteoporotic fracture by 30-70%.^8^ Therefore, international treatment guidelines have recommended that patients who experience a hip or vertebral fracture should receive AOMs to prevent future fracture.^1,9-11^ Meanwhile, in 2011, the Taiwanese Osteoporosis Association has launched their national guideline for osteoporosis treatment to suggest the use of AOMs in patient post osteoporotic fracture regardless of their BMD level.^12^ These recommendations were further supported by “real-world” evidence. For example, several nationwide ecologic studies have showed the increase in the prescription rate of AOMs was associated with decreases in the incidence of osteoporotic fractures.^13-16^ Two studies using Taiwan’s NHIRD further demonstrate the importance of adherence and persistence of AOMs on the clinical benefits of osteoporosis managements.^17,18^

 In Taiwan, the National Health Insurance (NHI) have reimbursed AOMs for patients encountering major osteoporotic fractures since 1987 although calcitonin was the only agent available between 1987 and 2001. From 2000 to 2010, bisphosphonates have become the predominant AOMs (Figure S1, see Supplementary file 1). However, the advance in osteoporosis treatment has resulted in significant increase of medical expenditures. The statistics released by Taiwan’s National Health Insurance Administration (NHIA) showed that the spending on AOMs have increased 7.2 folds from 1999 to 2010.^15^In 2011, a regulation in reimbursement of AOMs was thus released by the NHIA which required an additional check-up of BMD for the reimbursement of AOMs. To be more detailed, patients who suffered a hip or vertebral fracture or those who previously fulfill the reimbursement criteria of AOMs would need an additional record with BMD T-score of less than -2.5 standard deviation to be eligible for the reimbursement of AOMs since 2011 (Figure S1).

 As Taiwan’s NHI is a single payer system, such regulation on the reimbursement of AOMs is expected to have impacts on the utilization of AOMs. As we learned from previous studies,^13,14^ the utilization of AOMs were strongly linked to clinical outcomes. It is very important to know whether such regulation on the reimbursement of AOMs actually reduce medical expenditures and whether such regulation has any impact on clinical outcomes of osteoporotic patients.

 Therefore, the aim of this nationwide study was thus to examine the impacts of the regulation on the reimbursement of AOMs by NHIA on the utilization of AOMs, clinical outcomes, and medical expenditures of patients with incident hip fractures by using the already exit and versatile NHIRD in Taiwan.

## Methods

###  Database

 We used the data from NHIRD between 2005 and 2017 in Taiwan. The NHIRD is population-based claims data of Taiwan’s mandatory NHI program and is maintained by the Health and Welfare Data Science Center of the Ministry of Health and Welfare. The NHIRD contains longitudinal claims that reflect healthcare resource use including ambulatory visits, hospital care and prescribed medications as well as medical costs. All claims data of approximately 25 million beneficiaries (~99% of the total population in Taiwan) during the period of January 1, 2005 to December 31, 2017, were used as our data source.

###  Study Cohort

 Patients who firstly hospitalized for hip fractures between 2006 and 2015 were extracted as our study cohort and defined as patients with incident hip fractures. The identified hip fracture admission needs to be any admission with a primary diagnosis code in ICD-9-CM codes: 820 and concomitantly with ICD-procedure code: 8152, 7935, and 7915.^20,21^ Corresponding ICD-10-CM codes were applied when identifying study diagnosis codes after year 2015 (Table S1). The date of the first admission (index admission) was defined as the cohort entry date. Patients younger than 50 years old, with a pathological fracture (ICD-9-CM: 7331), with any diagnosis of fracture (ICD-9-CM: 805.xx-829.xx) or who were prescribed with AOM within one year prior to cohort entry date, died during the index admission or without a discharge record were excluded.

###  Study Cohort Stratification and Baseline Characteristics

 According to a previous research, we found age and gender are the most pronounced factors associated with probability to initiate AOMs therapy.^22^ Therefore, we stratified our study population into different gender (female vs. male) and age group (50-64, 65-79, and older than 80) to mitigate the influence of different age and gender on study outcomes. In addition, we collected index hospital characteristics and patient characteristics which may have impacts on the initiation of AOMs. Characteristics collected were included hospital region, hospital level, urbanization level, income level, comorbidities and co-medications. Urbanization level was classified into seven categories (1 refer to the most urbanized area, and 7 means the least urbanized area) according to the definition established by Taiwan National Health Research Institutes (NHRI).^23,24^

###  Intervention - the Policy Regulation on Reimbursement of AOMs

 The intervention evaluated in this study is the policy of this regulation on the reimbursement of AOMs on 2011.

###  Outcomes Measurements 

####  The Utilization of Anti-osteoporosis Medications

 The proportion of patients receiving AOMs within 1 year post index fracture admission was evaluated. The AOMs included in this study were alendronate, zoledronate, ibandronate, risedronate, denosumab, raloxifene, teriparatide, and calcitonin.

####  Bone Mineral Density Test 

 The proportion of patients undergoing a BMD test within 3 months post cohort entry date was evaluated. We use order code: 33064B to identify the BMD tests in NHIRD.

####  Osteoporotic Fracture Related Healthcare Expenditures 

 Annual osteoporotic related healthcare expenditures were estimated within 3-years post cohort entry date. Osteoporotic related healthcare includes hospitalization or outpatient visits with a primary diagnosis code in ICD-9-CM-code: 820, 805, 806, 812, 813.^25^

####  Subsequent Osteoporotic Fracture Related Visit 

 The proportion of patient encountered subsequent osteoporotic fracture related visit within 3-years post cohort entry date was evaluated. The definition of subsequent osteoporotic fracture related visit was summarized from previous studies which addressed subsequent osteoporotic fractures and using claims data.^17,20,26-28^ (Table S2) In this study, we integrated these criteria and defined the subsequent osteoporotic fracture among patients with hip fracture by two algorithms. First, the record of any new osteoporotic fractures (spine, humeral and wrist), either using specific ICD-9-CM-Codes or ICD-9-OP codes (Table S3), in addition to index hip fractures was defined as a subsequent fracture related visit. Second, any hospitalization with a primary diagnosis in ICD-9-CM codes: 820, and concomitantly with any of the ICD-procedure codes: 8152, 7935, 7915, which were occurred beyond 2 weeks post the index hip fracture admission. Corresponding ICD-10-CM codes were applied when identifying diagnosis since 2015 (Table S1).

###  Statistical Analysis

 We adopted the interrupted time series design with segmented regression analyses to evaluate the impacts of the regulation on the reimbursement of AOMs on the utilization of AOMs, clinical outcome, and medical expenditures of patients with incident hip fracture. This interrupted time series study design with segmented regression model is a quasi-experimental design that can control for baseline secular trend and quantitively estimate the level and trend changes attributable to the policy intervention. The change in level means an immediate change at the beginning of the post-regulation period and the change in trend means the long-term difference of trend between the pre- and post-regulation period.^29,30^

 The study employed a before–after design with a pre-regulation period consisting of a 5-years control phase from the first quarter (Q1) of 2006 to the fourth quarter (Q4) of 2010, and a 5-years of post-regulation period from 2011 to 2015. We used the Durbin-Watson test to take into account the presence of autocorrelation. We further stratified our study population into different gender (female vs male) and age group (50-64, 65-79, and older than 80) to see whether the impacts of reimbursement regulation varied by gender and age. Noteworthy, patients aged 50-64, 65-79 and older than 80 represented those relative young elderly, moderately old elderly and very old elderly, respectively. By this way, we can represent our research outcomes of each groups under similar aging degree, and provided the explicit results of patients with different aging degree.

## Results

**Figure 1:**
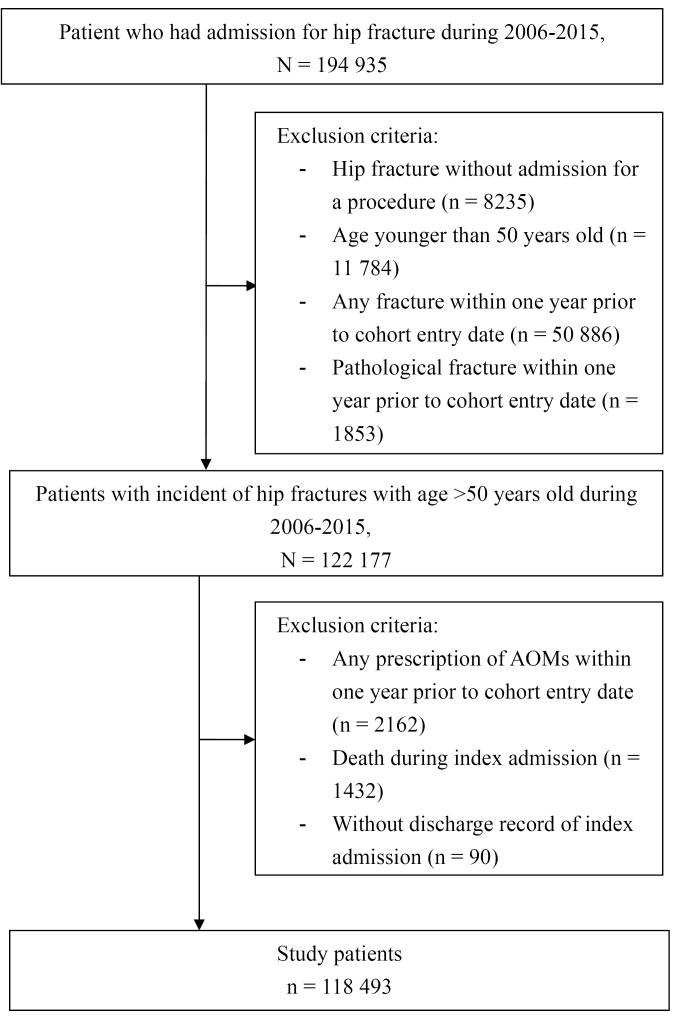
Study Flow Chart.

 After applying our inclusion and exclusion criteria, there were 118 493 patients aged more than 50 years old who were admitted for hip fracture during 2006-2015 (Figure 1). There were 7729, 28 831, and 33 408 female patients aged 50-64, 65-79, and 80 years or more, respectively. As for male patients, there were 8364, 18 817, and 21 344, in the corresponding age groups, respectively (Table 1). The index hospital characteristics and patient characteristics were similar within each identical age and gender strata (Table 1).

**Table 1:** Index Hospital Characteristics and Patient Characteristics of Each Age and Gender Strata

		**Pre-regulation Period (2006-2010)**						**Post-regulation Period (2011-2015)**					
		**Male**			**Female**			**Male**			**Female**		
*Variable*		**50-64**	**65-79**	**80+**	**50-64**	**65-79**	**80+**	**50-64**	**65-79**	**80+**	**50-64**	**65-79**	**80+**
Patient no. (n)		3819	10 124	9908	3559	14 679	15 508	4545	8693	11 436	4170	14 152	17 900
Hospital region, No. (%)	Taipei	1047 (27.4%)	2719 (26.9%)	3046 (30.7%)	1021 (28.7%)	4135 (28.2%)	5082 (32.8%)	1292 (28.4%)	2390 (27.5%)	3461 (30.3%)	1261 (30.2%)	3911 (27.6%)	5620 (31.4%)
	North	535 (14.0%)	1631 (16.1%)	1692 (17.1%)	519 (14.6%)	2071 (14.1%)	2230 (14.4%)	670 (14.7%)	1188 (13.7%)	1993 (17.4%)	573 (13.7%)	2048 (14.5%)	2658 (14.8%)
	Central	825 (21.6%)	1991 (19.7%)	1710 (17.3%)	685 (19.2%)	2951 (20.1%)	3010 (19.4%)	874 (19.2%)	1758 (20.2%)	1967 (17.2%)	779 (18.7%)	2719 (19.2%)	3417 (19.1%)
	South	1278 (33.5%)	3410 (33.7%)	3052 (30.8%)	1228 (34.5%)	5021 (34.2%)	4773 (30.8%)	1556 (34.2%)	3075 (35.4%)	3592 (31.4%)	1442 (34.6%)	5009 (35.4%)	5666 (31.7%)
	East	134 (3.5%)	373 (3.7%)	408 (4.1%)	106 (3.0%)	501 (3.4%)	413 (2.7%)	153 (3.4%)	282 (3.2%)	423 (3.7%)	115 (2.8%)	465 (3.3%)	539 (3.0%)
Hospital level	Medical center	1215 (26.7%)	2254 (25.9%)	2846 (24.9%)	1251 (30.0%)	3806 (26.9%)	4467 (25.0%)	1083 (28.4%)	2695 (26.6%)	2616 (26.4%)	1106 (31.1%)	4185 (28.5%)	4188 (27.0%)
	Regional hospital	2480 (54.6%)	4662 (53.6%)	6224 (54.4%)	2139 (51.3%)	7602 (53.7%)	9905 (55.3%)	1832 (48.0%)	4891 (48.3%)	4777 (48.2%)	1628 (45.7%)	6838 (46.6%)	7536 (48.6%)
	Local hospital	850 (18.7%)	1777 (20.4%)	2366 (20.7%)	780 (18.7%)	2744 (19.4%)	3528 (19.7%)	904 (23.7%)	2538 (25.1%)	2515 (25.4%)	825 (23.2%)	3656 (24.9%)	3784 (24.4%)
Urbanization	1 (Most urbanized)	1144 (30.0%)	2576 (25.4%)	2403 (24.3%)	1080 (30.3%)	4053 (27.6%)	4420 (28.5%)	1332 (29.3%)	2340 (26.9%)	2848 (24.9%)	1309 (31.4%)	3838 (27.1%)	4907 (27.4%)
	2	1603 (42.0%)	4708 (46.5%)	5022 (50.7%)	1620 (45.5%)	6485 (44.2%)	6862 (44.2%)	1921 (42.3%)	3713 (42.7%)	5498 (48.1%)	1795 (43.0%)	6251 (44.2%)	7686 (42.9%)
	3	331 (8.7%)	804 (7.9%)	699 (7.1%)	292 (8.2%)	1197 (8.2%)	1134 (7.3%)	435 (9.6%)	826 (9.5%)	944 (8.3%)	401 (9.6%)	1138 (8.0%)	1505 (8.4%)
	4	625 (16.4%)	1761 (17.4%)	1532 (15.5%)	513 (14.4%)	2593 (17.7%)	2705 (17.4%)	748 (16.5%)	1580 (18.2%)	1876 (16.4%)	594 (14.2%)	2559 (18.1%)	3339 (18.7%)
	5-6	43 (1.1%)	112 (1.1%)	103 (1.0%)	14 (0.4%)	135 (0.9%)	106 (0.7%)	51 (1.1%)	93 (1.1%)	105 (0.9%)	38 (0.9%)	147 (1.0%)	169 (0.9%)
	7 (Least urbanized)	73 (1.9%)	163 (1.6%)	149 (1.5%)	40 (1.1%)	216 (1.5%)	281 (1.8%)	58 (1.3%)	141 (1.6%)	165 (1.4%)	33 (0.8%)	219 (1.5%)	294 (1.6%)
Income	<US$700	1386 (36.3%)	4184 (41.3%)	5288 (53.4%)	1235 (34.7%)	4442 (30.3%)	4895 (31.6%)	1778 (39.1%)	2835 (32.6%)	5931 (51.9%)	1417 (34.0%)	4506 (31.8%)	5462 (30.5%)
	US$700-1000	1478 (38.7%)	4408 (43.5%)	3470 (35.0%)	1413 (39.7%)	7460 (50.8%)	7844 (50.6%)	1540 (33.9%)	3990 (45.9%)	4099 (35.8%)	1553 (37.2%)	6682 (47.2%)	9039 (50.5%)
	US$10 000+	955 (25.0%)	1532 (15.1%)	1150 (11.6%)	911 (25.6%)	2777 (18.9%)	2769 (17.9%)	1227 (27.0%)	1868 (21.5%)	1406 (12.3%)	1200 (28.8%)	2964 (20.9%)	3399 (19.0%)
Hypertension		1058 (27.7%)	4177 (41.3%)	3912 (39.5%)	1043 (29.3%)	7234 (49.3%)	7021 (45.3%)	1471 (32.4%)	3995 (46.0%)	5163 (45.1%)	1286 (30.8%)	7334 (51.8%)	9230 (51.6%)
Urinary Incontinence		6 (0.2%)	91 (0.9%)	115 (1.2%)	22 (0.6%)	205 (1.4%)	149 (1.0%)	17 (0.4%)	86 (1.0%)	143 (1.3%)	21 (0.5%)	173 (1.2%)	210 (1.2%)
Parkinson		44 (1.2%)	656 (6.5%)	646 (6.5%)	58 (1.6%)	790 (5.4%)	743 (4.8%)	77 (1.7%)	550 (6.3%)	874 (7.6%)	85 (2.0%)	781 (5.5%)	947 (5.3%)
Liver disease		353 (9.2%)	654 (6.5%)	362 (3.7%)	204 (5.7%)	929 (6.3%)	430 (2.8%)	401 (8.8%)	523 (6.0%)	444 (3.9%)	258 (6.2%)	845 (6.0%)	577 (3.2%)
Arthritis		124 (3.2%)	495 (4.9%)	446 (4.5%)	207 (5.8%)	1062 (7.2%)	816 (5.3%)	134 (2.9%)	420 (4.8%)	490 (4.3%)	242 (5.8%)	944 (6.7%)	852 (4.8%)
Autoimmune disease		Combined as 8			4 (0.1%)	11 (0.1%)	13 (0.1%)	Combined as 10		6 (0.1%)	7 (0.2%)	18 (0.1%)	17 (0.1%)
Catastrophic illness		592 (15.5%)	1596 (15.8%)	1205 (12.2%)	604 (17.0%)	2261 (15.4%)	1510 (9.7%)	697 (15.3%)	1515 (17.4%)	1505 (13.2%)	699 (16.8%)	2241 (15.8%)	1665 (9.3%)
Opiates		2110 (55.3%)	6695 (66.1%)	6530 (65.9%)	2395 (67.3%)	10 565 (72.0%)	10 218 (65.9%)	583 (12.8%)	1473 (16.9%)	1830 (16.0%)	563 (13.5%)	2596 (18.3%)	2471 (13.8%)
Non-opioid analgesics		2110 (55.3%)	6695 (66.1%)	6530 (65.9%)	2395 (67.3%)	10 565 (72.0%)	10 218 (65.9%)	2551 (56.1%)	5806 (66.8%)	7784 (68.1%)	2826 (67.8%)	10 190 (72.0%)	11 962 (66.8%)
Anxiolytics		1202 (31.5%)	4267 (42.1%)	4146 (41.8%)	1576 (44.3%)	7683 (52.3%)	7166 (46.2%)	1445 (31.8%)	3472 (39.9%)	4516 (39.5%)	1742 (41.8%)	7112 (50.3%)	8096 (45.2%)
Sedatives		718 (18.8%)	2497 (24.7%)	2460 (24.8%)	799 (22.5%)	4200 (28.6%)	3878 (25.0%)	829 (18.2%)	1883 (21.7%)	2570 (22.5%)	902 (21.6%)	3636 (25.7%)	4097 (22.9%)
Corticosteroids		906 (23.7%)	3114 (30.8%)	2917 (29.4%)	959 (26.9%)	4264 (29.0%)	3493 (22.5%)	1068 (23.5%)	2672 (30.7%)	3360 (29.4%)	1201 (28.8%)	4317 (30.5%)	4191 (23.4%)

**Figure 2:**
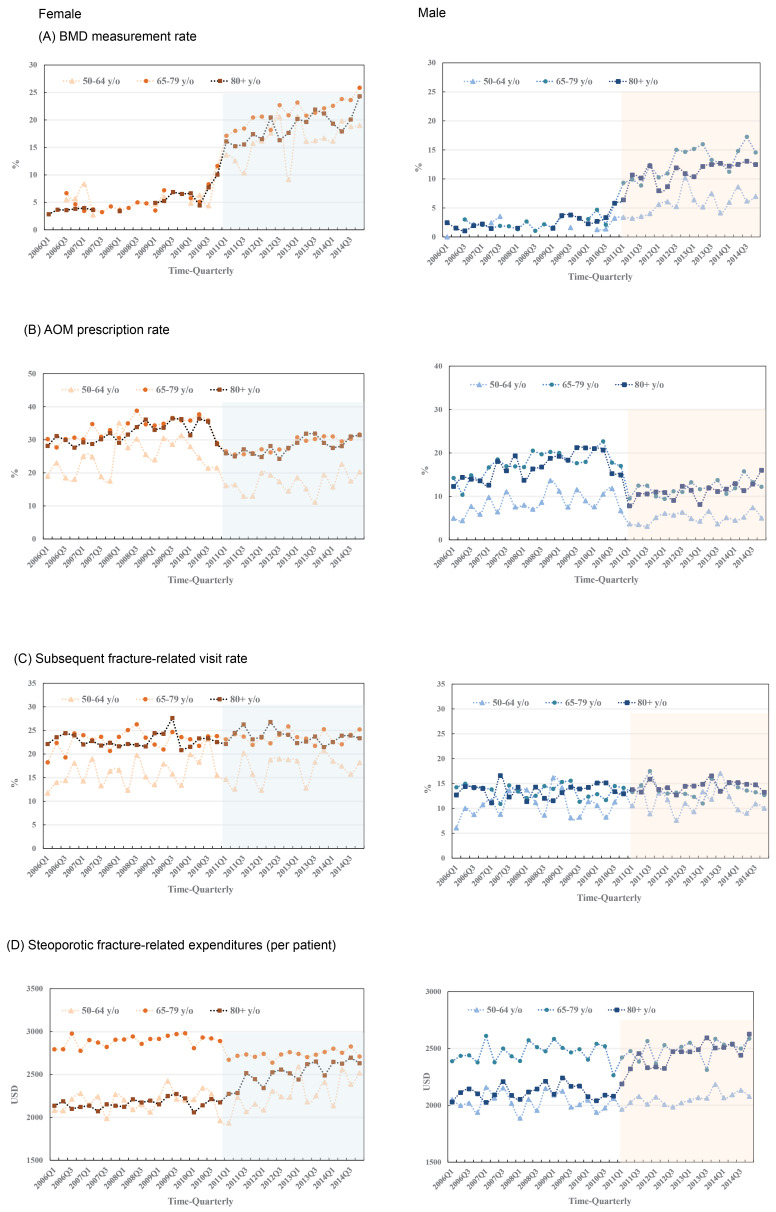
Changes in National Health Insurance Reimbursement Criteria on (A) BMD measurement within 3 months post index hip fracture, (B) AOM prescription rate within 1 year post index hip fracture, (C) Subsequent osteoporotic fracture-related visit within 3 years post index hip fracture, and (D) Osteoporotic fracture-related healthcare expenditures within 3 years post index hip fracture per patient. Shadow means the period post reimbursement criteria change since January 2011. Abbreviations: BMD, bone mineral density; AOMs: anti-osteoporosis medications.

 The proportion of patients who underwent BMD test post index hip fracture significantly increased immediately and continuously since the change of reimbursement criteria in 2011 (Figure 2, Table 2). The increased trends were found in every gender and age groups, and the exact rate increased from less than 5% in the first quarter (Q1) of 2006 to nearly 25% and 20% in the last quarter (Q4) of 2017 for female and male patients, respectively. The largest increase in the first several quarters of post-regulation period were seen in female patients aged between 65 and 79 years (Figure 2, Table 2).

**Table 2:** Trend and Level Changes of Study Outcomes Due to Reimbursement Changes

	**BMD Exam**		**AOMs Prescriptions**		**Subsequent Fracture**		**Fracture Related Cost**	
	**Level Change** **%, (95% CI)**	**Trend Change** **%, (95% CI)**	**Level Change** **%, (95% CI)**	**Trend Change** **%, (95% CI)**	**Level Change** **%, (95% CI)**	**Trend Change** **%, (95% CI)**	**Level Change** **USD, (95% CI)**	**Trend Change** **USD, (95% CI)**
Female, age (y)								
50-64y	6.5*** (3.8, 9.2)	0.3** (0.1, 0.5)	-13.0*** (-18.0, -7.7)	-0.2 (-0.6, 0.3)	-2.5 (-5.5, 0.4)	-0.3 (-0.3, 0.3)	-188* (-335, -40)	23** (9, 37)
65-79	10.5*** (8.8, 12.3)	0.2** (0.1, 0.4)	-5.6 (-10.2, -0.9)	-0.3 (-0.8, 0.2)	-0.4 (-2.7, 1.9)	-0.1 (-0.3, 0.1)	-249*** (-304, -194)	1 (-4, 6)
+80	8.0*** (5.7, 10.2)	0.2* (0.1, 0.4)	-6.8** (-10.7, -2.9)	-0.2 (-0.6, 0.1)	1.4 (-0.9, 3.7)	-0.1 (-0.3, 0.1)	139** (55, 224)	19*** (11, 27)
Male, age (y)								
50-64	2.0* (0.1, 4.0)	0.2** (0.1, 0.4)	-5.7*** (-8.1, -3.4)	-0.2 (-0.4, 0.0)	0.2 (-3.5, 4.0)	-0.1 (-0.5, 0.3)	-29 (-118, 60)	9* (0.1, 17)
65-79	6.5*** (4.4, 8.5)	0.2** (0.1, 0.4)	-8.9*** (-12.3, -5.6)	-0.3* (-0.6, -0.0)	1.1 (-0.9, 3.2)	-0.0 (-0.2, 0.2)	-64 (-150, 23)	6 (-2, 14)
+80	4.7*** (3.1, 6.2)	0.3** (0.1, 0.4)	-9.2*** (-12.9, -5.6)	-0.2 (-0.5, 0.1)	0.1 (-1.3, 1.5)	0.0 (-0.1, 0.2)	168*** (76, 260)	18*** (9, 27)

Abbreviations: AOMs, anti-osteoporosis medications; BMD, bone mineral density. *** *P* ≤.001, ** *P *≤.01, * *P* ≤.05.

 During the pre-regulation period, the prescription rate of AOMs within one year post index hip fracture was approximately 20% and 40% among male and female patients, respectively. Since 2011, the prescription rate significantly declined in all sex and age groups, except for female patients aged 65 and 79 years. The largest decreases were seen in female patients aged 50 and 64 years and male patients older than 65 years. Notably, the declines were immediate while being temporarily, and only continued among male patients aged 65-79 years. The interrupted time analysis showed no statistically difference in trend changes (Figure 2, Table 2). There were no significant differences in the proportions of patients who have subsequent visits for osteoporotic fracture between the pre- and post-regulation periods.

 Regarding the osteoporotic fracture-related healthcare expenditures post hip fracture, we found different patterns among patients younger or older than 80 years. Among patients aged 50-79 years, an immediate decrease in medical expenditures was found, while an increase in medical expenditures were observed in the long-term trend among those aged 50-64 years. On the contrary, an immediate increase in fracture related expenditures among patients older than 80 years post regulation was noted. Besides, there was a continued and significant increase among male patients aged 80 years and older. This means that in the post-regulation period, male patients older than 80 years having hip fracture spent more money on fracture-related expenditures than did those with hip fracture before regulation and the increase in costs continued.

## Discussion

 This nationwide study evaluated the “real-world” impacts of the regulation on the reimbursement of AOMs since 2011. This evaluation revealed that the regulation in reimbursement substantially influenced patients who encountered hip fracture, especially their post fracture utilization of AOMs and osteoporotic related expenditures. Besides, the impact varied by different age and gender groups of patients. The impact of the regulation on the reimbursement on the utilization of AOMs was immediate and temporary.

 The BMD examination rate measured in this study was a surrogate of the physicians’ awareness to initiate AOMs. In other words, when physician arranged BMD exam for their patients, it means that thees physicians were intended to prescribe AOMs to treat their patients. Therefore, we found the rate of BMD examination was associated with the rate of prescription of AOMs. Before 2011, the examination rate of BMD was less than 5%, and the rate increased to almost 25% at the last quarter of 2015, which indicating the impact of this regulation in reimbursement as it requires additional BMD check to meet the eligibility of reimbursement of AOMs. In other words, we could reasonably presume that a quarter of the physicians were aware of the importance of AOMs for their patients and arranged the BMD examination as a preparation for that. However, according to the consensus of international treatment guidelines, patients encountering hip fracture were recommended to initiate AOM therapy regardless of their BMDs.^1,15-17^If those patients who underwent BMD examination were expected to initiate AOMs according to guidelines, it means there were 75% of candidates for AOMs not being arranged to received their osteoporosis treatments.

 Even the increase in rates of BMD measurement was observed in this study, if these patients’ BMD levels were not low enough, they still cannot receive their AOMs. For instance, among female patients older than 65 years, their BMD examination rate was similar to their treatment rate of AOMs; however, among female patients younger than 65 years, though their BMD examination rate was nearly 25% in 2015, their AOM treatment rate was less than 20%. One reasonable speculation is that almost 5% of female patients younger than 65 years did not have BMD low enough to initiate AOMs. However, among female patients aged 65 years or older, the BMD examination rate and AOM prescription rate were comparable, both being nearly 25%. A similar finding was observed in the male population, except that their proportion did not reach the BMD criteria were higher than that of female population.

 The prescription rate of AOM decreased immediately, especially among patients younger than 65 years old and older than 80 years old. We found that the decrease mainly occurred immediately at the post-regulation period and its trend was diminished one year after the post-regulation period. However, there was a gap observed in the AOM prescription rate between the pre- and post-regulation periods. Results of the BMD examination provide sufficient evidence to initiate AOMs, therefore, the difference between BMD examination rate and actual AOMs prescription rate could be expected to be due to the “insufficiency” of BMD loss among patients younger than 65 years old. Consequently, BMD level became another obstacle to receiving AOMs, in addition to physician’s awareness of the need to initiate AOMs. Besides, patients older than 80 years old may due to the ambulation or lower accessibility to hospital revisit and results in decrease of the utilization of AOMs. Compared to patients younger than 65 years old and older than 80 years old, those aged 65-79 were relative unaffected by BMD and physical restriction. Due to the very unchangeable property of our NHI reimbursement criteria of AOMs, the improvement of physicians’ awareness of the need to initiate AOMs is one of the most practical and critical way to enhance the overall osteoporosis therapy. However, the optimization of osteoporotic fracture therapy is still implementation of the well-established evidence-based treatment in our daily practice through reimbursement criteria. For example, every patient should accomplish their BMD examination and assess the appropriateness of AOMs during their index hospitalization for the hospital to acquire NHI reimbursement. Thus, patients who need the treatment could initiate AOM therapy immediately on their first return to the outpatient department.

 We examined the rate of subsequent osteoporotic fracture-related visits; however, we did not find significant difference in changes in both levels and trends between patients who encountered hip fracture during the pre- and post-regulation periods. There are several explanations for this. First, we found that when we used the subsequent fracture as the outcome variable, the coefficient of determination (R^2^) of the segmented regression models were very low (range from 0.05 and 0.27, please see Table S4). In other words, the two main variables in the regression model, the reimbursement regulation on AOMs on 2011 and the time frame, cannot well predict the risk of subsequent osteoporotic fracture.^31^ This is very reasonable, because the risk of fracture is multifactorial and there are confounders that could not be captured in our claims database. For example, the usage of AOMs at patient’s own expense can also influence the risk of subsequent fracture. Second, we found the adherence of AOMs of patients encountered hip fracture post the AOMs restrain policy on 2011 was significantly increased than that before the policy intervention. Therefore, the clinical effectiveness of AOMs therapy may become better among patients with higher adherence. Third, although we did not evaluate the individual AOMs use pattern among study population, but due to previous market survey, the market shares of long-acting AOMs (such as denosumab, and zoledronate) were stably increased through years. By this way, the adherence of AOMs therapy was simultaneously increased. Notably, although there were no significant differences in rate of subsequent fracture, there still is an increasing trend, especially among patients aged 80 years or older, and the impact was revealed through the increase in osteoporotic fracture-related expenditures.

 Interestingly, a significant increase in costs both immediately and in the long-term pattern were observed among patients aged 80 years or older. We tried to conduct further analyses and found several potential reasons for this. First, we found that the adherence and persistence of AOMs therapy increased among the patients who encountered hip fracture post 2011; therefore, the total cost of AOMs per patient increased. Second, the reimbursement change resulted in delay or decrease in AOM therapy and induced the poor control over complications following an incident hip fracture, which consumed more healthcare resources. Therefore, the frequency of osteoporotic fracture-related visits per person increased significantly (Table S5 and Table S6). Meanwhile, the proportion of patients who encountered subsequent osteoporotic fracture-related visit increased among those who were the most susceptible and the oldest study population.

 To our knowledge, this study is the first to examine the real world impact of the regulation in reimbursement of AOMs. This study has several merits. First, we performed a comprehensive assessment of the natural treatment process of patients who encountered hip fracture. Second, by adopting the interrupted time series study design, we could eliminate the influence of baseline secular time trend and figure out the impact of the regulation on short-term changes in the level and long-term trend changes. Third, by using the NHIRD, this study was nationally representative and had a longitudinal follow-up of 3 years. Finally, this study directly measured the real-world impact of the regulation in reimbursement from the perspective of our NHIA and provided informative and unbiased results for policy-makers to optimize the reimbursement policy for patients with osteoporotic fracture.

**Figure 3:**
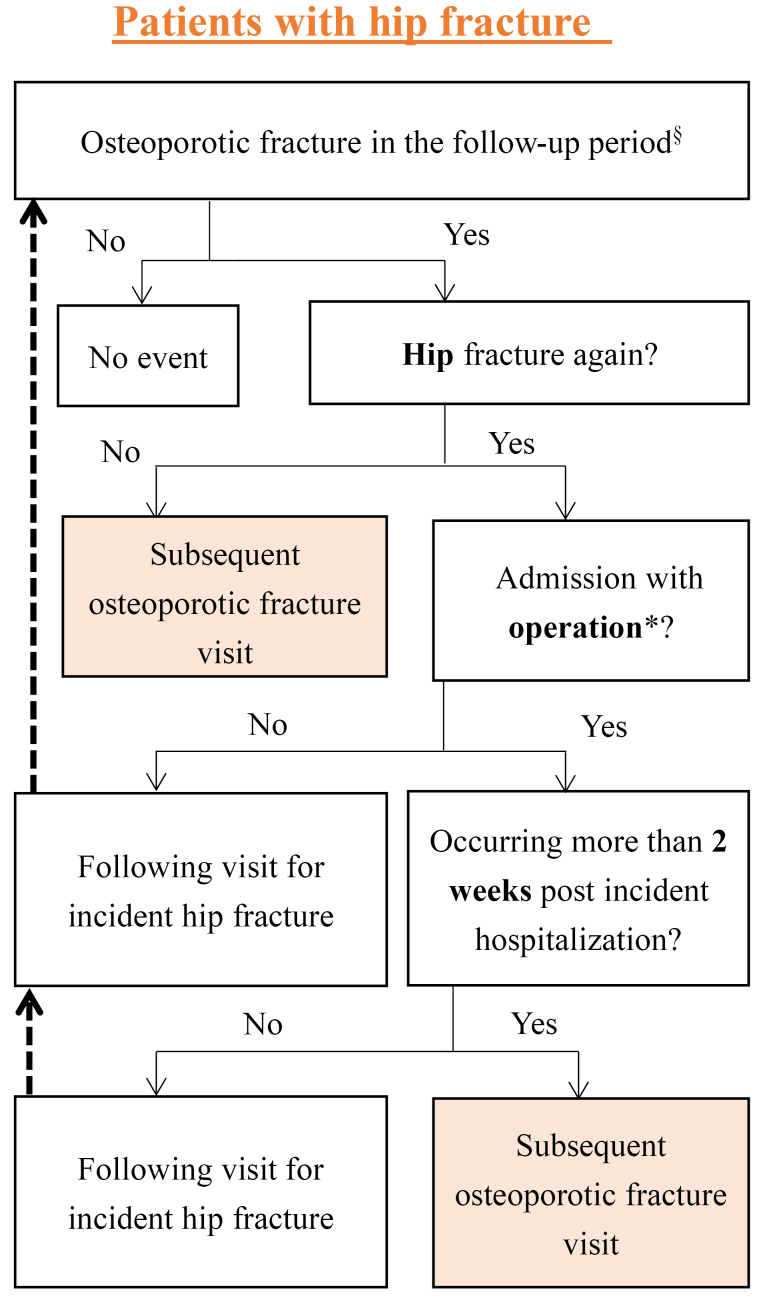
Algorithm for Define Subsequent Osteoporotic Fracture Related Visit by Using Claims Data. § Osteoporotic fracture means hip, spine, humeral and wrist fracture and detect by primary diagnose code in ICD-9-codes: hip = 820, spine = 805 or 806, humeral = 812, wrist = 813, or ICD-OP-codes: hip = 81.52; 79.35; 79.15; spine = 03.53; 78.49; humeral = 78.52; 79.01; 79.11; 79.21; 79.31, wrist = 78.53; 79.02; 79.12; 79.22; 79.32. * ICD-OP-codes: hip = 81.52; 79.35; 79.15.

 However, there are several limitations that need to be declared. First, the identifying of subsequent osteoporotic fracture is relatively difficult in claims data. However, we have reviewed previous studies to generate practically and clinically relevant algorithms (Figure 3). In addition, we modified the term of subsequent osteoporotic fracture into subsequent osteoporotic fracture-related visit to make our operational definition more precise. Second, as mentioned above, the status of out-of-pocket usage of AOMs cannot be captured in our claims-based study. Besides, some factors related to the utilization of AOMs did not record in the NHIRD, therefore we cannot make discussions on it. For instance, some patients did not receive the AOMs. This may be due to their drug allergy history, ambulation status or even the doubt of benefit of AOMs by the physician or patient themselves. Finally, in order to make sure the validity of our inclusion of study cohort, patients who didn’t receive hip fracture related surgery (nearly 4.2%) were not included in this study. Noteworthy, those did not receive surgery may due to very old age and severe co-morbidities, which may cause more profound medical costs. However, this deficit cannot overshadow the importance of our findings about the exact increase in medical expenditures related to osteoporotic fractures during the post-regulation period, especially from the perspective of NHIA.

## Conclusion

 The additional requirement of BMD evidences for the reimbursement of AOMs on 2011 decreased the prescribing rate of AOMs immediately although the effect did not sustain thereafter. However, higher subsequent osteoporotic fracture related medical expenditures were introduced, especially among those very old population. Therefore, the timely initiation of AOMs therapy post hip fracture was important and BMD may not be an appropriate decisive criteria. Re-evaluating this reimbursement criteria is highly recommended.

## Acknowledgements

 We thank the Taiwan NHIA and NHRI for making their databases available; however, this article in no way represents any official position of the NHIA or the NHRI.

## Ethical issues

 The study protocol was approved by the Research Ethics Committee of National Taiwan University Hospital (NTUH REC-201812019W) and informed consents were waived.

## Competing interests

 Authors declare that they have no competing interests.

## Authors’ contributions

 Study design: CYW, SHF, and FYH; Performed research: CYW, CCH, and JWL; Analysed data: CYW and HMC; Contributed new methods or models; RSY, FYH, and LJS; Wrote the paper: CYW, SHF, FYH, and LJS.

## Funding

 This study was supported by the Research Assistantships funded by the Ministry of Science and Technology, Taiwan, [grant number MOST 104-2410-H-002-225-MY3, to FYH]; National Taiwan University Hospital Yun-Lin Branch [grant number NTUHYL108.S016, to CCH]; and National Taiwan University Hospital Yun-Lin Branch [grant number NTUHYL106.A001, to JWL]. CYW was supported by a scholarship from the XinChen Medical Research Foundation, Taiwan.

## Authors’ affiliations

^1^School of Pharmacy, College of Medicine, National Taiwan University, Taipei, Taiwan. ^2^Graduate Institute of Clinical Pharmacy, College of Medicine, National Taiwan University, Taipei, Taiwan. ^3^Department of Pharmacy, National Taiwan University Hospital Yun-Lin Branch, Douliu, Taiwan. ^4^Department of Orthopedics, National Taiwan University Hospital Yun-Lin Branch, Douliu, Taiwan. ^5^Department of Orthopedics, National Taiwan University Hospital, Taipei, Taiwan. ^6^Cardiovascular Center, National Taiwan University Hospital Yun-Lin Branch, Douliu, Taiwan. ^7^Health Data Research Center, National Taiwan University, Taipei, Taiwan. ^8^Department of Pharmacy, National Taiwan University Hospital, Taipei, Taiwan

## Supplementary files


Supplementary file 1 contains Figure S1 and Tables S1-S6.
