# Supplementary material for: Impact of the Requirement of Bone Mineral Density Evidence on Utilization of Anti-osteoporosis Medications, Clinical Outcome and Medical Expenditures of Patient With Hip Fracture in Taiwan
Source: Int J Health Policy Manag. 2020 Oct 3;11(4):470–8. doi: 10.34172/ijhpm.2020.169 (PMC9309953; doi:10.34172/ijhpm.2020.169)
Supplement: Supplementary file 1 — contains Figure S1 and Tables S1-S6. [file ijhpm-11-470-s001.pdf]

Supplementary file 1

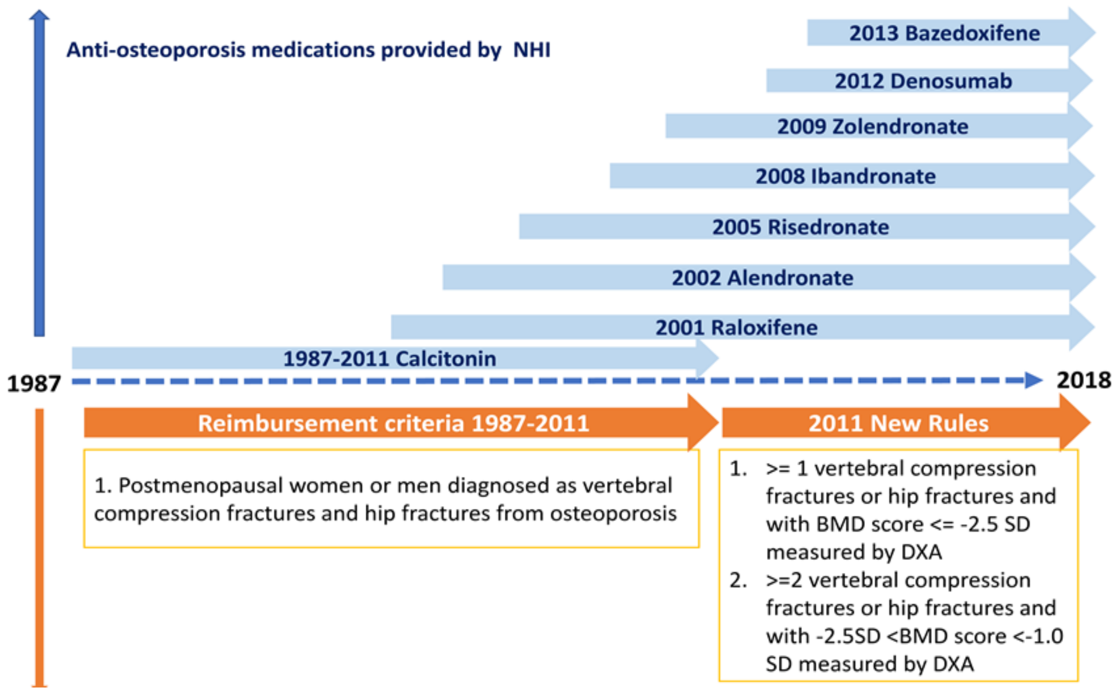

Figure S1. Reimbursement of Anti-osteoporosis Medications in Taiwan Since 1987.

Table S1. ICD-9-Codes Used in This Study and the Corresponding ICD-10-CM Codes Applied When Identifying Study Diagnosis Codes After Year 2015

ICD-9-Codes	ICD-10-Codes
Hip 820	S72.0, S72.1
Spine 805, 806	S22, S32
Humeral 812	S42
Wrist 813	S52
ICD-9 OP-Codes	ICD-10-OP-Codes
Hip Fracture	
81.52	0SRA009,0SRA00A,0SRA00Z,0SRA019,0SRA01A,0SRA01Z,0SRA039,0SRA03A,0SRA03Z,0SRA07Z,0SRA0J9,0SRA0JA,0SRA0JZ,0SRA0KZ,0SRE009,0SRE00A,0SRE00Z,0SRE019,0SRE01A,0SRE01Z,0SRE039,0SRE03A,0SRE03Z,0SRE07Z,0SRE0J9,0SRE0JA,0SRE0JZ,SRE0KZ,0SRR019,0SRR01A,0SRR01Z,0SRR039,0SRR03A,0SRR03Z,0SRR07Z,0SRR0J9,0SRR0JA,0SRR0JZ,0SRR0KZ,0SRS019,0SRS01A,0SRS01Z,0SRS039,0SRS03A,0SRS03Z,0SRS07Z,0SRS0J9,0SRS0JA, 0SRS0JZ,0SRS0KZ
79.35	0QS604Z,0QS606Z,0QS704Z,0QS706Z
79.15	0QS634Z,0QS644Z,0QS734Z,0QS744Z,0QS834Z,0QS844Z,0QS934Z,0QS944Z,0QSB34Z, 0QSB44Z,0QSC34Z,0QSC44Z
Vertebral Fracture	
03.53	0PS304Z, 0PS30ZZ, 0PS334Z, 0PS33ZZ, 0PS344Z, 0PS34ZZ, 0PS3XZZ, 0PS404Z, 0PS40ZZ, 0PS434Z, 0PS43ZZ, 0PS444Z, 0PS44ZZ, 0PS4XZZ, 0QS004Z, 0QS00ZZ, 0QS034Z,0QS03ZZ, 0QS044Z, 0QS04ZZ, 0QS0XZZ, 0QS104Z, 0QS10ZZ, 0QS134Z, 0QS13ZZ, 0QS144Z, 0QS14ZZ, 0QS1XZZ, 0PU307Z, 0PU30JZ, 0PU30KZ, 0PU337Z, 0PU33JZ, 0PU33KZ, 0PU347Z, 0PU34JZ, 0PU34KZ, 0PU407Z, 0PU40JZ, 0PU40KZ, 0PU437Z, 0PU43JZ, 0PU43KZ, 0PU447Z, 0PU44JZ, 0PU44KZ, 0QU007Z, 0QU00JZ, 0QU00KZ, 0QU037Z, 0QU03JZ, 0QU03KZ, 0QU047Z, 0QU04JZ,<br>0QU04KZ, 0QU107Z, 0QU10JZ, 0QU10KZ, 0QU137Z, 0QU13JZ,<br>0QU13KZ, 0QU147Z, 0QU14JZ, 0QU14KZ
78.49	0PQ30ZZ, 0PQ33ZZ, 0PQ34ZZ, 0PQ3XZZ, 0PQ40ZZ, 0PQ43ZZ, 0PQ44ZZ, 0PQ4XZZ, 0QQ00ZZ, 0QQ03ZZ, 0QQ04ZZ, 0QQ0XZZ, 0QQ10ZZ, 0QQ13ZZ, 0QQ14ZZ, 0QQ1XZZ, 0PU307Z, 0PU30JZ,0PU30KZ, 0PU337Z, 0PU33JZ, 0PU33KZ, 0PU347Z, 0PU34JZ, 0PU34KZ, 0PU407Z, 0PU40JZ, 0PU40KZ, 0PU437Z, 0PU43JZ, 0PU43KZ, 0PU447Z, 0PU44JZ, 0PU44KZ, 0QU007Z, 0QU00JZ, 0QU00KZ, 0QU037Z, 0QU03JZ, 0QU03KZ, 0QU047Z, 0QU04JZ, 0QU04KZ, 0QU107Z, 0QU10JZ, 0QU10KZ, 0QU137Z, 0QU13JZ,0QU13KZ, 0QU147Z, 0QU14JZ,

	0QU14KZ, 0PS304Z, 0PS30ZZ, 0PS334Z, 0PS33ZZ, 0PS344Z, 0PS34ZZ, 0PS3XZZ, 0PS404Z, 0PS40ZZ, 0PS434Z, 0PS43ZZ, 0PS444Z, 0PS44ZZ, 0PS4XZZ, 0QS004Z, 0QS00ZZ, 0QS034Z, 0QS03ZZ, 0QS044Z, 0QS04ZZ,0QS0XZZ, 0QS104Z, 0QS10ZZ, 0QS134Z, 0QS13ZZ, 0QS144Z, 0QS14ZZ, 0QS1XZZ, 0PU307Z, 0PU30JZ, 0PU30KZ, 0PU337Z,0PU33JZ, 0PU33KZ, 0PU347Z, 0PU34JZ, 0PU34KZ, 0PU407Z, 0PU40JZ, 0PU40KZ, 0PU437Z, 0PU43JZ, 0PU43KZ, 0PU447Z, 0PU44JZ, 0PU44KZ, 0QU007Z, 0QU00JZ, 0QU00KZ, 0QU037Z, 0QU03JZ, 0QU03KZ, 0QU047Z, 0QU04JZ, 0QU04KZ, 0QU107Z, 0QU10JZ, 0QU10KZ, 0QU137Z, 0QU13JZ, 0QU13KZ, 0QU147Z, 0QU14JZ, 0QU14KZ
Humeral	
79.01	0PSC3ZZ,0PSC4ZZ,0PSCXZZ,0PSD3ZZ,0PSD4ZZ,0PSDXZZ
79.11	0PSC34Z,0PSC36Z,0PSC44Z,0PSC46Z,0PSD34Z,0PSD36Z,0PSD44Z,0PSD46Z
79.21	0PSC0ZZ,0PSD0ZZ
79.31	0PSC04Z,0PSC06Z,0PSD04Z,0PSD06Z
Wrist	
78.53	0PHH04Z,0PHH34Z,0PHH44Z,0PHJ04Z,0PHJ34Z,0PHJ44Z,0PHK04Z,0PHK34Z,0PHK44Z,0PHL04Z,0PHL34Z,0PHL44Z
79.02	0PSH3ZZ,0PSH4ZZ,0PSHXZZ,0PSJ3ZZ,0PSJ4ZZ,0PSJXZZ,0PSK3ZZ,0PSK4ZZ,0PSKXZZ,0PSL3ZZ,0PSL4ZZ,0PSLXZZ
79.12	0PSH34Z,0PSH36Z,0PSH44Z,0PSH46Z,0PSJ34Z,0PSJ36Z,0PSJ44Z,0PSJ46Z,0PSK34Z,0PSK36Z,0PSK44Z,0PSK46Z,0PSL34Z,0PSL36Z,0PSL44Z,0PSL46Z
79.22	0PSH0ZZ,0PSJ0ZZ,0PSK0ZZ,0PSL0ZZ
79.32	0PSH04Z,0PSH06Z,0PSJ04Z,0PSJ06Z,0PSK04Z,0PSK06Z,0PSL04Z,0PSL06Z

Table S2. Studies Evaluating AOMs Effectiveness and Defining Subsequent Osteoporotic Fracture as Outcomes by Using Claims Data

**Author, year, country**	**Study population**	**Clinic outcomes**	**Outcome Definition**	**Criteria adopted in the study**				
				**ICD**	**Visit type**	**Time**	**OP**	**Exam**
Ryg et al.<br>2009 Denmark,	Incidence of Hip fracture	Secondary hip fracture	1. The fractures were validated by matching fracture date with date of surgical procedure codes<br>2. Patients referred from out-patient clinics were excluded	○	○	○	○	
Overman et al<br>2015, USA,	AOMs initiator	Osteoporotic fractures	1. Hip, pelvis, humerus, wrist - used ICD-9 code with ICD-9-OP codes;<br>2. Spine fractures - used ICD-9 codes	○			○	
Hawley et al<br>2016, UK,	Hip Fracture	Subsequent major re-fracture/<br>Hip re-fracture	1. Sustaining a subsequent major fracture within 3 years of the primary event.<br>2. Hip fractures were only included if sustained between 6 and 36 months so as to avoid counting of re-coding events.	○		○		
Author<br>(year, country, journal)	Study population	Clinic outcomes	Outcome Definition	Criteria adopted in the study				
				ICD	Visit type	Time	OP	Exam
Lin et al, 2013,Taiwan	HIP/Spine fractures + initiating AOMs	Non vertebral fractures<br>(Hip, humerus, or radius fractures)	1. All outcomes were derived from inpatient claims.	○	○			
Lee et al, 2016, Taiwan	Hip Fracture	Secondary hip	1. Fractures occurring more than 2 weeks after the first fracture<br>2. Validated as an incident hip fracture: (ICD-9-CM:	○	○	○	○	

			820 with ICD-9-CM Procedure Code 81.52 or internal fixation: 79.15, 79.35).					
Soong et al, 2013, Taiwan	AOMs initiator	Risk of re-fracture/ Probable osteoporotic fracture	1. Hospitalization and outpatient department surgical procedures for a new osteoporotic fracture during follow-up.<br>2. Hospitalizations were selected based on discharge diagnosis of probable osteoporotic fracture	○	○		○	

Abbreviations: AOMs, Anti-osteoporosis medication; OPD, outpatient department; ICD, ICD-9-CM Codes; Visit type, admission or OPD; Time, consider time interval or temporal relationship; OP, operation; Exam, fracture related radiological imaging examination.

Table S3. Specific Codes for Identifying Osteoporotic Fractures

	Hip	Spine	Humeral	Wrist
ICD-9-CM code	820	805 or 806	812	813
ICD-9-OP codes	81.52; 79.35; 79.15	08.53; 78.49	87.52; 79.01; 79.11; 79.21; 79.31;79.61	78.53; 79.02; 79.12; 79.22; 79.32; 79.62

Table S4. Coefficient of determination (R^2) and results of Durbin-Watson test for each segmented regression model

	**BMD exam**		**AOMs prescriptions**		**Subsequent fracture**		**Fracture related cost**	
	**Model: R square**	**Durbin-Watson**	**Model: R square**	**Durbin-Watson**	**Model: R square**	**Durbin-Watson**	**Model: R square**	**Durbin-Watson**
Female, age (y)								
50-64	0.87	1.90	0.62	1.96	0.27	2.06	0.42	2.07
65-79	0.98	1.97	0.69	2.07	0.18	1.89	0.77	2.00
+80	0.97	1.96	0.60	1.95	0.11	1.96	0.91	2.01
Male, age (y)								
50-64	0.82	1.98	0.59	2.06	0.06	1.93	0.16	1.99
65-79	0.94	1.98	0.78	1.85	0.05	1.98	0.21	2.05
+80	0.96	1.93	0.76	1.96	0.20	1.85	0.91	1.99

Table S5. Osteoporotic fracture related visits within 3 years post index hip fracture

	**Female-Osteoporotic fracture related visits (Frequency)**								
	**Age 50-64**			**Age 65-79**			**Age 80+**		
	**OPD**	**Hosp**	**ER**	**OPD**	**Hosp**	**ER**	**OPD**	**Hosp**	**ER**
2006Q1	939	194	44	3554	822	226	3256	871	301
2006Q2	1069	212	53	3502	789	259	2651	764	268
2006Q3	938	161	51	3221	761	224	3064	742	223

	Female-Osteoporotic fracture related visits (Frequency)								
	Age 50-64			Age 65-79			Age 80+		
	OPD	Hosp	ER	OPD	Hosp	ER	OPD	Hosp	ER
2006Q4	932	198	63	4027	864	294	3561	861	264
2007Q1	962	181	56	4226	917	290	3283	920	291
2007Q2	1111	195	59	3782	708	260	2403	705	231
2007Q3	835	149	42	3486	786	239	2961	757	231
2007Q4	1041	189	62	4462	907	292	3280	915	272
2008Q1	1493	227	63	4058	956	304	3800	978	312
2008Q2	1051	182	56	3769	759	262	3376	795	257
2008Q3	1212	178	66	4394	824	277	3131	784	266
2008Q4	1370	225	74	4244	904	310	3593	922	309
2009Q1	1371	206	63	4334	911	376	4311	1031	342
2009Q2	1144	165	48	3562	738	269	3297	815	314
2009Q3	1509	188	64	3748	773	246	4090	858	311
2009Q4	1535	194	67	3933	858	325	4324	955	360
2010Q1	1240	209	80	4136	812	378	3977	955	440
2010Q2	1453	190	89	3200	699	301	3757	858	389
2010Q3	1344	204	85	3929	778	341	3614	801	334
2010Q4	1661	218	80	3932	784	319	3943	956	398
2011Q1	1554	220	76	4732	901	334	5643	1140	437
2011Q2	1512	204	79	4344	759	261	3888	788	297
2011Q3	1384	207	88	4015	752	240	3766	809	302
2011Q4	1633	236	62	4959	827	284	4779	998	396
2012Q1	1816	255	82	5345	907	333	5783	1132	410
2012Q2	1409	191	62	3941	669	232	4572	823	263
2012Q3	1572	208	63	4460	738	240	4218	870	282
2012Q4	1166	200	60	4437	825	271	4639	1029	351
2013Q1	1453	211	72	4895	839	270	6047	1141	414
2013Q2	1503	229	71	3946	666	195	4561	847	284
2013Q3	1314	215	48	4821	744	260	5147	918	329
2013Q4	1683	255	82	5407	823	285	6062	1107	345
2014Q1	1809	273	86	5607	878	324	6649	1200	411

	Female-Osteoporotic fracture related visits (Frequency)								
	Age 50-64			Age 65-79			Age 80+		
	OPD	Hosp	ER	OPD	Hosp	ER	OPD	Hosp	ER
2014Q2	1756	246	63	4021	703	227	4254	896	323
2014Q3	1277	193	79	4237	769	270	4749	927	333
2014Q4	1957	283	100	4683	881	321	5734	1150	425

Table S6. Osteoporotic Fracture Related Visits Within 3 Years Post Index Hip Fracture

	Male-Osteoporotic fracture related visits (Frequency)								
	Age 50-64			Age 65-79			Age 80+		
	OPD	Hosp	ER	OPD	Hosp	ER	OPD	Hosp	ER
2006Q1	726	187	46	2753	680	196	2122	523	144
2006Q2	994	188	42	2462	543	171	1633	431	137
2006Q3	887	191	54	2354	540	177	1389	416	110
2006Q4	830	198	67	2496	629	182	1974	504	176
2007Q1	779	172	44	2215	567	176	2354	581	204
2007Q2	811	181	55	2103	503	146	2274	452	161
2007Q3	1078	212	42	1789	515	155	1669	462	144
2007Q4	1038	210	61	2403	588	164	2464	563	168
2008Q1	764	185	56	2670	648	220	2230	644	182
2008Q2	898	196	47	2187	531	159	1557	480	170
2008Q3	925	198	55	1816	542	164	1511	469	146
2008Q4	1190	220	57	2852	604	214	2394	653	230
2009Q1	1153	233	70	2830	619	196	2685	677	251
2009Q2	825	208	54	2026	498	176	2227	522	175
2009Q3	1013	197	49	2322	507	159	2368	463	161
2009Q4	1159	236	61	2395	531	187	3013	629	210
2010Q1	1578	260	120	2929	597	248	3033	650	299
2010Q2	1404	251	104	2303	464	233	2142	562	230
2010Q3	1231	209	67	2323	481	198	2401	520	210
2010Q4	1041	239	74	2328	490	202	2798	655	239
2011Q1	1654	242	97	2904	562	210	3580	766	295
2011Q2	1396	247	85	2764	524	178	2200	539	205
2011Q3	1218	249	79	2052	456	144	2552	517	179
2011Q4	1375	237	82	2231	513	159	3019	620	213
2012Q1	1557	227	68	1880	441	172	2474	547	183
2012Q2	1329	256	75	2111	468	154	2351	520	181

	**Male-Osteoporotic fracture related visits (Frequency)**								
	**Age 50-64**			**Age 65-79**			**Age 80+**		
	**OPD**	**Hosp**	**ER**	**OPD**	**Hosp**	**ER**	**OPD**	**Hosp**	**ER**
2012Q3	1173	240	75	2586	466	158	3162	703	222
2012Q4	1491	267	92	3044	490	156	3306	681	263
2013Q1	1379	252	87	2461	471	133	2585	552	194
2013Q2	1367	241	77	2164	447	153	2847	564	199
2013Q3	1347	264	90	2330	472	145	3701	774	263
2013Q4	1388	304	75	2245	505	157	3685	746	241
2014Q1	1159	242	61	1993	445	158	2844	564	174
2014Q2	1295	253	70	2238	441	136	2962	565	187
2014Q3	1322	284	89	2265	471	167	3667	702	251
2014Q4	1557	227	68	1880	441	172	2474	547	183
